# Impact of Cold Ischemic Time and Freeze-Thaw Cycles on RNA, DNA and Protein Quality in Colorectal Cancer Tissues Biobanking

**DOI:** 10.7150/jca.29372

**Published:** 2019-08-27

**Authors:** Xin-Juan Fan, Yan Huang, Pei-Huang Wu, Xin-Ke Yin, Xi-Hu Yu, Xin-Hui Fu, Li-Li Feng, Yun-Long Wang, Hong-Jun Yi, Zhi-Ting Chen, Jun-Xiang Yin, Da-Lu Zhang, Wei-Xing Feng, Shao-Mei Bai, Taewan Kim, Gordon B. Mills, Yi-Ling Lu, Xiang-Bo Wan, Lei Wang

**Affiliations:** 1 Guangdong Institute of Gastrointestinal, Guangzhou, Guangdong, China.; 2 Department of Pathology, the Sixth Affiliated Hospital of Sun Yat-sen University, Guangzhou, Guangdong, China.; 3 Department of Gastrointestinal Surgery, the Sixth Affiliated Hospital of Sun Yat-sen University, Guangzhou, Guangdong, China.; 4 China National Center for Biotechnology Development, Beijing, China.; 5 The Ohio State University Comprehensive Cancer Center, Columbus, Ohio, USA.; 6 Department of Systems Biology, Division of Basic Science Research, The University of Texas MD Anderson Cancer Center, Houston, TX, USA.; 7 Department of Radiation Oncology, the Sixth Affiliated Hospital of Sun Yat-sen University, Guangzhou, Guangdong, China.; XJ Fan, Y Huang, PH Wu and XK Yin contributed equally to this study.

**Keywords:** colorectal tissue bank, quality control, RNA integrity, DNA integrity, protein expression.

## Abstract

Tissue-derived RNA, DNA and protein samples become more and more crucial for molecular detection in clinical research, personalized and targeted cancer therapy. This study evaluated how to biobanking colorectal tissues through examining the influences of cold ischemic time and freeze-thaw cycles on RNA, DNA and protein integrity. Here, 144 pairs of tumor and normal colorectal tissues were used to investigate the impact of cold ischemic times (0-48h) on RNA, DNA and protein integrity at on ice or room temperature conditions. Additionally, 45 pairs of tissues experienced 0-9 freeze-thaw cycles, and then the RNA, DNA and protein quality were analyzed. On ice, RNA, DNA and protein from colorectal tumor and normal tissues were all stable up to 48h after surgery. At room temperature, RNA in colorectal tumor and normal tissues began to degrade at 8h and 24h, respectively. Meanwhile, the tumor tissues DNA degradation occurred at 24h after surgery at room temperature. Similarly, the protein expression level of tumor and normal tissues began to change at 24h after the surgery at room temperature. Interestingly, tissue RNA and DNA remained stable even after 9 freeze-thaw cycles, whereas the proteins levels were remarkably changed after 7 freeze-thaw cycles. This study provided a useful evidence on how to store human colorectal tissues for biobanking. Preserving the surgical colorectal tissue on ice was an effective way to prevent RNA, DNA and protein degradation. Importantly, more than 7 repeated freeze-thaw cycles were not recommended for colorectal tissues.

## Introduction

Tissue-derived RNA, DNA and protein samples have become more essential for molecular analysis in medical research, personalized and targeted cancer therapy 1. The accuracy of microarray and genomic analyses largely relies on the availability and quality of the biobank-preserved biospecimens 2. The biomedical research conducted by low-quality RNA, DNA and protein could cause a serious distortion of the credibility of the research. Thus, it is indispensable to standardize the biobanking protocols to obtain a high quality specimen for reliable fundamental and translational research.

The importance of specimens in the translational cancer research has enhanced the foundation and development of tissue banks in lots of medical centers 3. However, the protocols for sample processing and storage are varied among different institutional biobanks. And the non-standardized protocols give rise to the inconsistency in the quality of tissue-derived samples, and consequently, lead to the varied results among the different biomedical analyses. It is known that the inconsistency is mainly caused by surgical procedures, such as cold ischemic time, size of the tissue, temperature during delivery, and freeze-thaw cycles.

In clinical practice, cold ischemic time and freeze-thaw cycles have been considered as the key factors to determine the RNA, DNA and protein quality of tissues 4, 5. Indeed, prolonged cold ischemic time led to the RNA degradation in breast cancers tissue samples 6. A recent study profiling a number of proteins in renal carcinoma tissues, and showed that different ischemic time conditions dramatically modify the protein expression signature 7. Consistently, several studies had confirmed that prolonged cold ischemia time lowered the tissue quality, and was the pivotal cause of protein degradation 8, 9. In addition, repeated freeze-thaw cycles also resulted in tissue destruction and deteriorated quality of RNA, DNA and protein. For instance, Jochumsen et al. found that RNA quality and quantity in ovarian tumor tissues could be diminished after 3 cycles of freezing and thawing 10. Therefore, it is extremely urgent to develop and establish the standardized biobanking protocols for each tissue subtype.

In this study, we developed a standard procedure of tissue biobanking for colorectal cancer (CRC) patients, particularly in terms of cold ischemic time and freeze-thaw cycles by examining RNA, DNA and protein integrity in various conditions. We found that the quality of RNA, DNA and protein remain stable up to 48h after surgery if tissues were stored on ice. On the contrary, RNA of tissues left at room temperature was degraded at 8h after surgery. Additionally, the tumor tissues DNA began to degrade at 24h after surgery, and the protein expression pattern in colorectal tumor and normal tissues began to change at 24h after the surgery at room temperature. Furthermore, for both tumor and normal tissues, RNA and DNA remained stable even after 9 freeze-thaw cycles, whereas the proteins expression levels were remarkably changed after 7 freeze-thaw cycles.

## Materials and Methods

### CRC tissue bank

The Tissue Bank of the Sixth Affiliated Hospital, Sun Yat-sen University, as the biggest colorectal tissue bank in Southern China, coupled with a comprehensive clinical database and follow-up system, was established in 2007. Here, 112,140 colorectal tissue samples had been collected from 6,230 patients who underwent a radical CRC surgery from January 2007. For each case, 18 fresh tissue samples would be stored at the biobank, including of 9 tumor tissues and 9 paired normal adjacent tissues. The written patients consent for tissue banking was approved before the surgery for each case.

### Tissue biobanking process

After resection, the specimens were transferred into pre-chilled cryovials and stored at on ice immediately, followed by delivering to the tissue biobank within 10 min after the surgery. Well trained research assistants were responsible for the initial gross examination. Subsequently, the tissues were then cut into pieces with the dimension of 0.5*0.5*0.5 cm^3^ for bio-banking. Moreover, the normal mucosa at 5cm far away from the tumor was collected simultaneously. Half part of the tumor and the paired normal adjacent tissues were then moved to labelled cryovials, and stored in a ultra-low freezer (-80℃). Additionally, another half part of the tumor and paired normal adjacent tissue samples were incubated in the RNAlater stabilization reagents (Ambion Inc., Austin, TX, USA) at 4℃ overnight, and further stored in the ultra-low freezer for long-term storage. Clinical information and tissues ex-vivo ischemic time were precisely recorded for each sample.

### Study design

**Figure 1:**
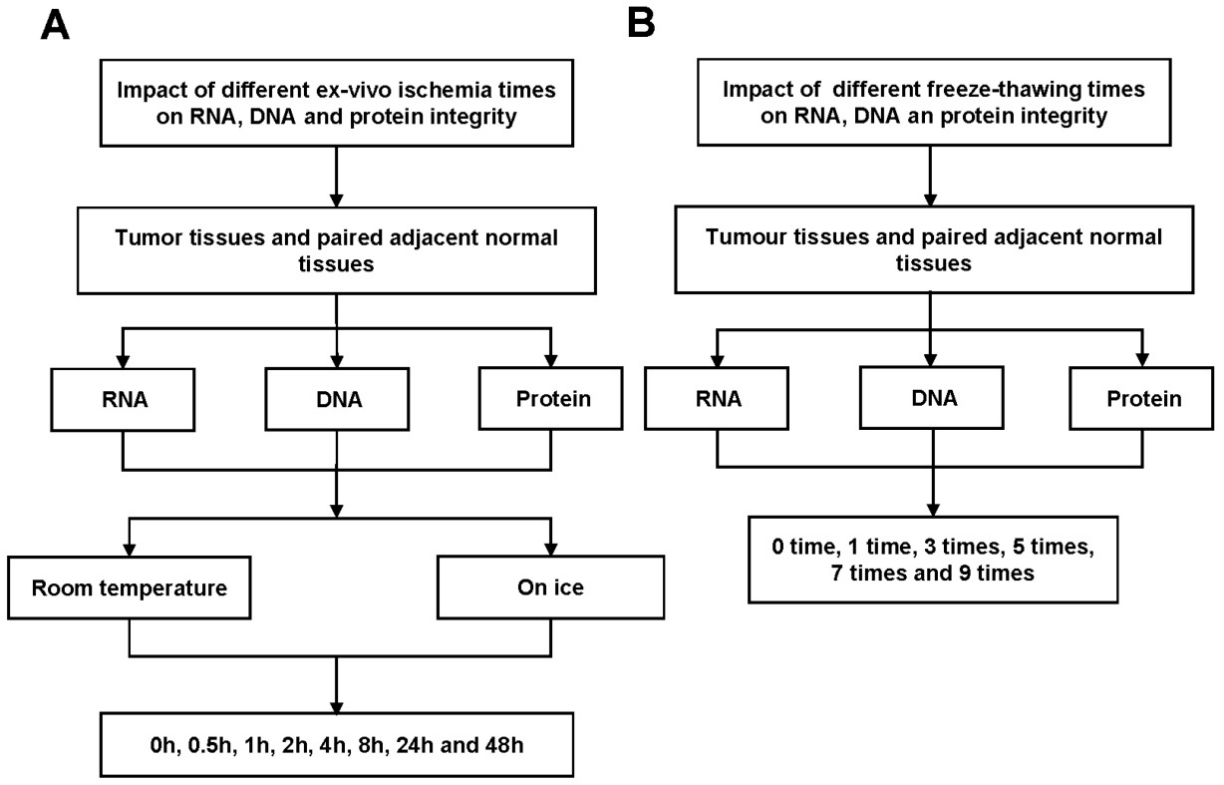
** Workflow for RNA, DNA and protein quality assessment.** (A) Tumor tissues and paired adjacent normal tissues not only used to detect the effect of cold ischemia times (0h, 0.5h, 1h, 2h, 4h, 8h, 24h and 48h) on RNA, DNA and protein quality kept at room temperature or on ice. (B) but also used to evaluate the impact of repeated freeze-thaw cycles (1 time, 3 times, 5 times, 7 times and 9 times).

In this study, we examined the effects of different cold ischemia time (0h, 0.5h, 1h, 2h, 4h, 8h, 24h and 48h) at on ice or room temperature (Figure 1A and Figure S1A), and freeze-thaw cycles (1 time, 3 times, 5 times, 7 times and 9 times, Figure 1B and Figure S1B) on RNA, DNA and protein integrity for CRC and normal adjacent tissues. The study was approved by the Clinical Ethics Review Committee at the Sixth Affiliated Hospital of Sun Yat-sen University.

### Tissue cold ischemic times

To analyze the impact of tissue cold ischemic time on RNA, DNA and protein quality, fresh tumor and paired normal adjacent tissues were collected. For each case, the tumor and normal adjacent tissues were obtained immediately after surgical resection in the operating room. The tissue was divided into 16 pieces with equal size. Half subgroup (8 pieces) was placed at room temperature (25℃), while another half subgroup (8 pieces) was placed on ice. One piece in each subgroup was immediately immersed into liquid nitrogen, and then transferred to -80℃ for further analysis and considered as 0h. After 0.5h, 1h, 2h, 4h, 8h, 24h and 48h, one piece was taken from each subgroup to extract the RNA, DNA and protein immediately at the end of each time point. For RNA analysis, the tissues were always immersed in RNAlater stabilization reagents to prevent RNA degradation.

### Repeated freeze-thaw cycles

To investigate the effect of repeated freeze-thaw times on tissue RNA, DNA and protein integrity, 45 pairs of CRC and normal tissues were tested (Figure S1B). Each sample was divided into 5 pieces to analyze the integrity of RNA, DNA and protein. All of the samples were then transferred into -80℃ ultra-low freezer. At least 4h after the freezing, all the samples were taken from the -80℃ ultra-low freezer and placed on ice until the tissues were completely thawed. This process was considered as 1 cycle of freeze-thaw.

### RNA extraction and quality analysis

Tissue RNA extraction was performed using RNeasy Mini Kit (Qiagen Inc, Valencia, CA, USA) and RNase-Free DNAase Set (Qiagen Inc., Valencia, CA, USA) in accordance with the manufacturer's instructions. The isolated RNA was analyzed by Agilent 2100 Bioanalyzer (Version A.02 S1292, Agilent Technologies, Waldbronn, Germany). To assess the RNA integrity, RNA integrity numbers (RINs) were calculated by Agilent software, and RIN ≥ 7.0 was considered as high-quality and intact RNA samples from the tissues.

### DNA extraction and quality analysis

DNA was extracted and purified using Tissue gDNA kit (Biomiga Inc., San Diego, CA, USA) and Wizard Genomic DNA Purification kit (Promega Corp., Madison, WI, USA), according to the manufacturer's instructions. DNA concentration was estimated by measuring the absorbance at 260 nm. DNA integrity was determined by agarose gel electrophoresis. Equal amount of extracted DNA for each sample was loaded to 1.5% agarose gel and stained with ethidium bromide for imaging.

### Protein extraction and reverse phase protein array (RPPA) analysis

In this study, the RPPA approach was used to analyze the protein expression levels at RPPA core facility of the University of Texas MD Anderson Cancer. Colorectal tumor and paired normal adjacent tissues were lysed in RPPA lysis buffer [1% Triton X-100, 50mM HEPES (pH 7.4), 150mM NaCl, 1.5mM MgCl_2_, 1mM EGTA, 100mM NaF, 10mM NaPPi, 10% glycerol supplemented with fresh PMSF (1mM final concentration), Na_3_VO_4_ (1mM final concentration), protease (cOmplete Tablets EDTA-free) and phosphatase (PhosSTOP) inhibitor cocktail (Roche Applied Science)]. Tumor and normal adjacent tissue lysates were serially diluted two-fold for 5 dilutions (from undiluted to 1:16 dilution) and printed on nitrocellulose-coated slides using an Aushon Biosystems arrayer (Burlington, MA) in an 11x11 format. Samples were stained with primary and secondary antibodies in an autostainer (BioGenex), probed by tyramide-based signal amplification approach and then visualized by DAB colorimetric reaction. RPPA slides were stained for 298 unique antibodies (Table S1), which were analyzed on Array-Pro, then by SuperCurve Rx64 3.1.1. Relative protein expression levels for each sample were determined by interpolation of each dilution curve from the "standard curve" (supercurve) of the slide (antibody). Supercurve was constructed by a script in R, written by Bioinformatics. Then slides were scanned on a flatbed scanner to produce 16-bit tiff image. Spots from tiff images were identified and the density was quantified by Array-Pro Analyzer. R packages were used for all statistical analyses (version 2.10.0).

## Results

### Impact of cold ischemic times on colorectal tissue RNA integrity

**Figure 2:**
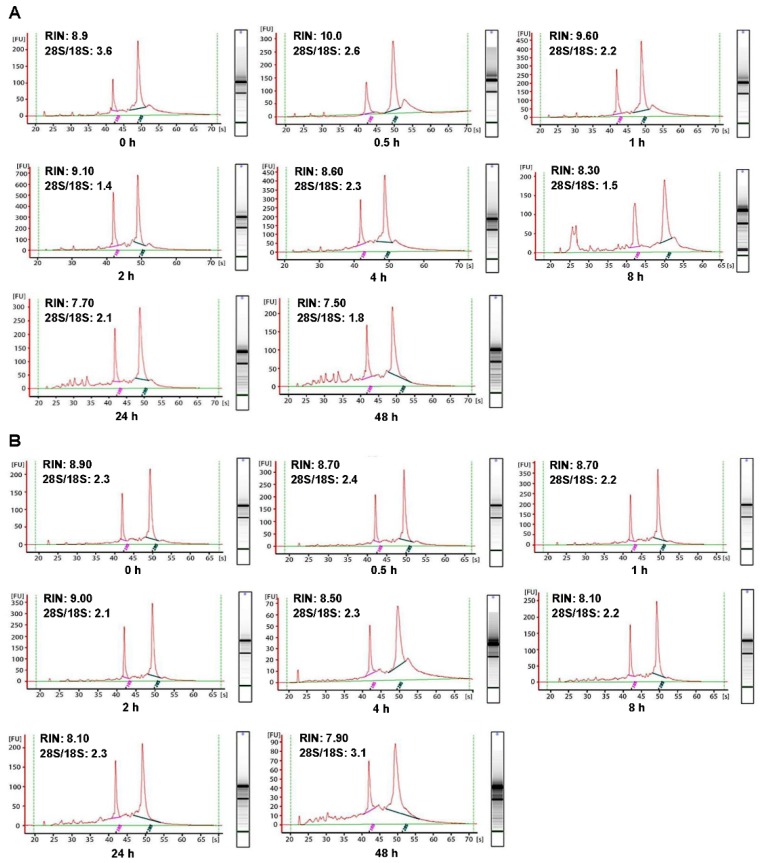
** Impact of different cold ischemic times on RNA integrity of colorectal tumor and normal tissues when placed at on ice.** (A) The RIN of tumor tissues when placed on ice at different hours after surgery. (B) The RIN of normal tissues when placed on ice at different hours after surgery. The RIN of all samples was larger than 7.0.

**Figure 3:**
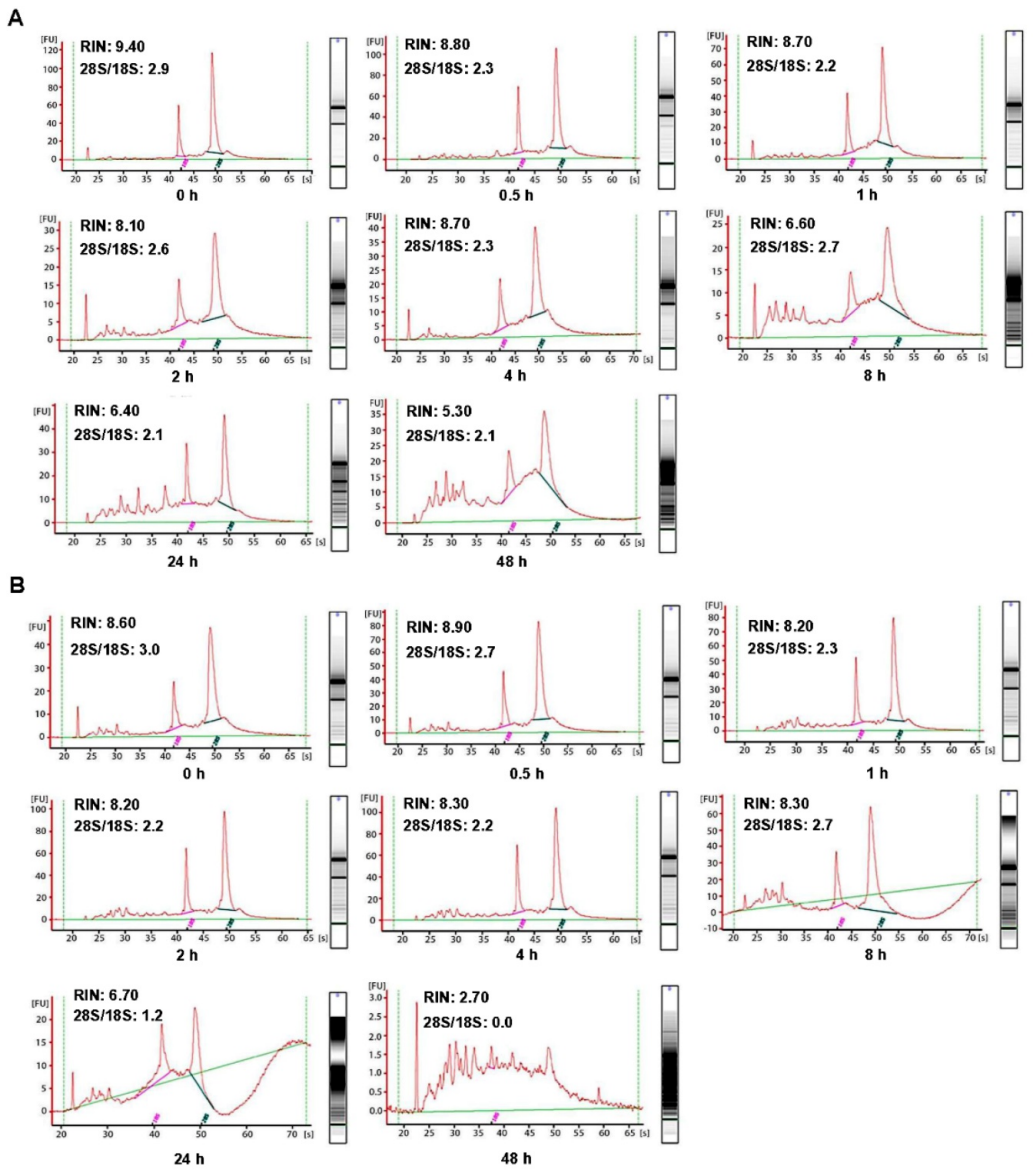
** Impact of different cold ischemic times on RNA integrity of colorectal tumor and normal tissues when preserved at room temperature.** (A) The RIN of tumor tissues when placing at room temperature was decreased to 6.6 at 8h, and the value of RIN lower than 7.0 after 8h. (B) The RIN of normal tissues when placing at room temperature was decreased to 6.7 at 24h, and the value of RIN lower than 7.0 after 24h.

In order to investigate the influence of cold ischemic time on RNA quality, we analyzed 48 pairs of CRC and normal adjacent tissues. Both tumor tissues (Figure 2A) and normal tissues (Figure 2B) presented ribosomal peaks up to 48h when the tissues were placed on ice. For tumor tissues, the mean RNA Integrity Numbers (RINs) were 8.5, 9.5, 8.8, 9.2, 8.8, 8.7, 7.3 and 7.5 at 0h, 0.5h, 1h, 2h, 4h, 8h, 24 and 48h after the surgery (with all *P*> 0.05), respectively. For normal tissues the mean RINs were 8.8, 8.7, 8.9, 8.2, 8.5, 8.1, 7.8 and 7.7 at 0h, 0.5h, 1h, 2h, 4h, 8h, 24h and 48h after the surgery (with all *P*> 0.05), respectively. These results indicated that all tissue RNA with a good quality when placed at on ice. By contrast, when the tissues were processed at room temperature (25℃), the tumor tissues RNA (Figure 3A) was degraded at 8h, and normal tissues RNA (Figure 3B) degradation was shown at 24h. The mean RIN was decreased to 5.7 at 8h for the tumor tissues and to 4.4 at 24h for the normal tissues (data not shown), suggesting that the quality of tissues was aggravated and not suitable for further research usage.

### Impact of cold ischemic times on colorectal tissue DNA integrity

**Figure 4:**
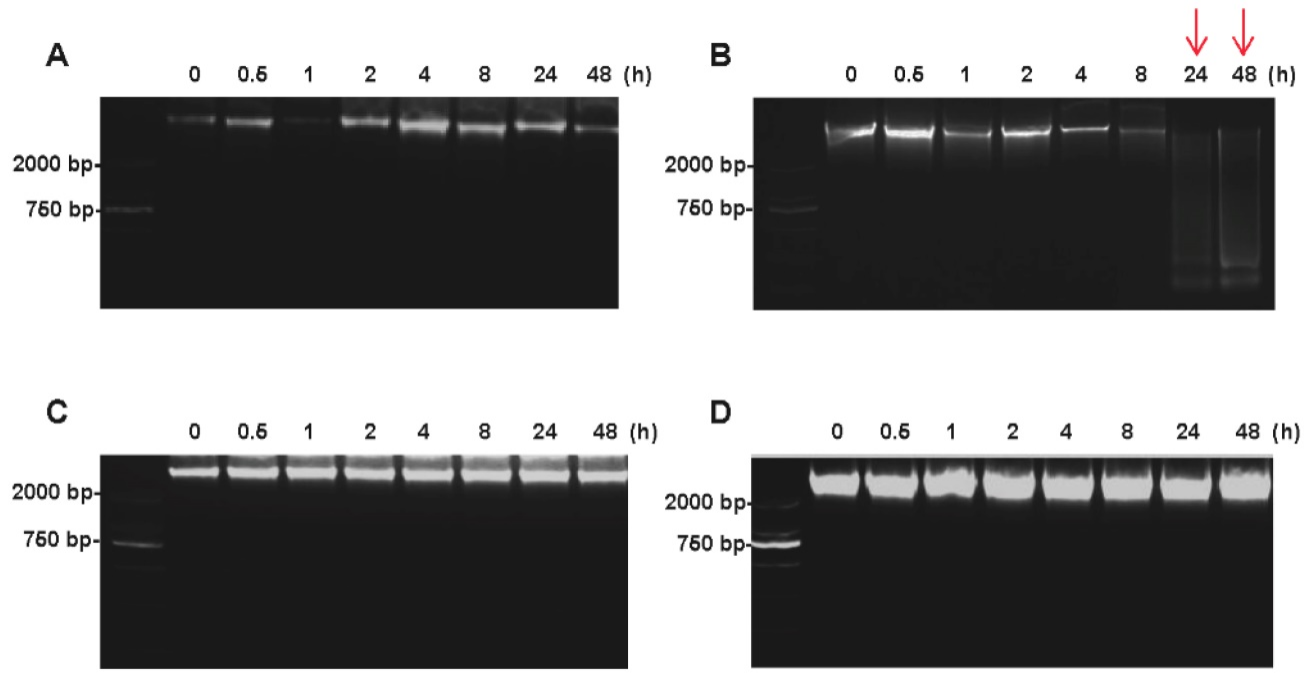
** Impact of different cold ischemic times on tumor and normal tissue DNA integrity when placed at on ice or room temperature.** (A) Electropherogram of tumor tissues DNA at different hours on ice. (B) Electropherogram of tumor tissues DNA at different hours on room temperature. (C) Electropherogram of normal tissues DNA at different hours on ice. (D) Electropherogram of normal tissues DNA at different hours on room temperature. At room temperature, the DNA degradation was observed at 24h in tumor tissues, but not in normal tissues.

The DNA integrity of colorectal tissues was determined by agarose gel electrophoresis. For tumor tissues, the high-molecular-weight bands were found at the top of the DNA ladder up to 48h on ice (Figure 4A), whereas the smeared low-molecular-weight DNA was observed when the tumor tissues were kept at room temperature for 24h (Figure 4B). For normal tissues, no obvious difference was found between at on ice and room temperature up to 48h after the surgery (Figure 4C and 4D).

### Impact of cold ischemic times on colorectal tissue protein expression pattern

**Figure 5:**
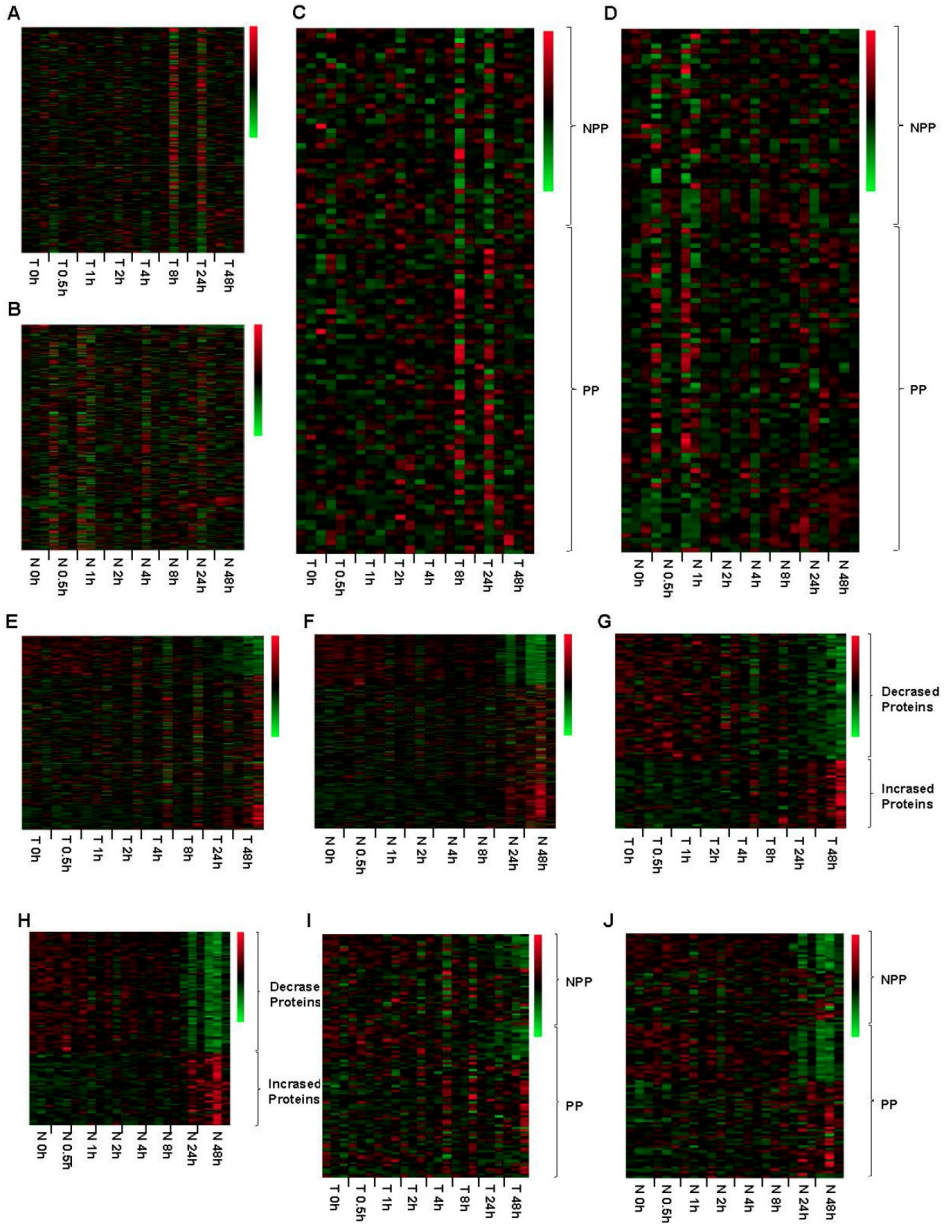
** Impact of different cold ischemic times on tumor and normal tissues' 298 proteins expression when kept at ice or room temperature.** For tumor (A) and normal tissues (B), the protein expression level was stable when placed at on ice for 48h after the surgery. Green color in the heatmap indicates the expression of protein is low, and red color in the heatmap indicates the protein are overexpression. The expression pattern of phosphorylated proteins (PP) and part of non-phosphorylated proteins (NPP) was stable up to 48h after the surgery (C and D). At room temperature, the protein expression levels were obviously changed both in tumor (E) and normal tissues (F) from 48h after the surgery. Totally, 60 proteins were decreased expressed and 32 proteins were increased expressed in tumor tissues (G). Moreover, 79 proteins were decreased expressed and 47 proteins were increased expressed in paired normal tissues (H). Among these proteins, 13 phosphorylated proteins were decreased expressed and 2 phosphorylated proteins were increased expressed in the tumor tissues (I), and 22 phosphorylated proteins were decreased expressed and 7 phosphorylated proteins was increased expressed in the paired normal tissues (J). T 4h, T indicated Tumor tissue, and 4h indicated that the tumor tissue was placed at on ice or room temperature for 4h. N 48h, N indicated Normal tissue, and 48h indicated that the normal tissue was placed at on ice or room temperature for 48h.

RPPA method had been proved to be a powerful, high-throughput, quantitative, and cost-effective technology for functional proteomics studies. In this study, RPPA was used to detect the expression level of 298 proteins, including of 65 phosphorylated proteins (Table S1). Up to 48h after the surgery, none of the tested proteins in both tumor and normal tissues showed any significant expressional changes when tissues were kept on ice (Figure 5A and 5B). Specifically, the expression levels of phosphorylated proteins, which are thought to be degraded easily, was also stable up to 48h after surgery when kept on ice (Figure 5C and 5D). When the tissues were processed at room temperature, the protein expression levels were obviously changed at 48h (Figure 5E and 5F). For tumor and normal tissues, we found that 60 and 79 proteins expression levels were decreased, while other 32 and 47 proteins expression levels were increased at 24h after the surgery, respectively (Figure 5G and 5H, Table S2). Similarly, the phosphorylated proteins expression levels were also changed at 24h. For tumor tissues, 14 phosphorylated proteins were decreased expressed, and other 2 phosphorylated proteins were increased expressed at 24h (Figure 5I, Table S3). The phosphorylated proteins in normal adjacent tissues showed a similar changing pattern, including of 22 phosphorylated proteins were decreased expressed and 7 were increased expressed at 24h (Figure 5J, Table S3), suggesting that phosphorylated proteins are susceptible to temperature.

### Impact of freeze-thaw cycles on RNA, DNA and protein integrity

**Figure 6:**
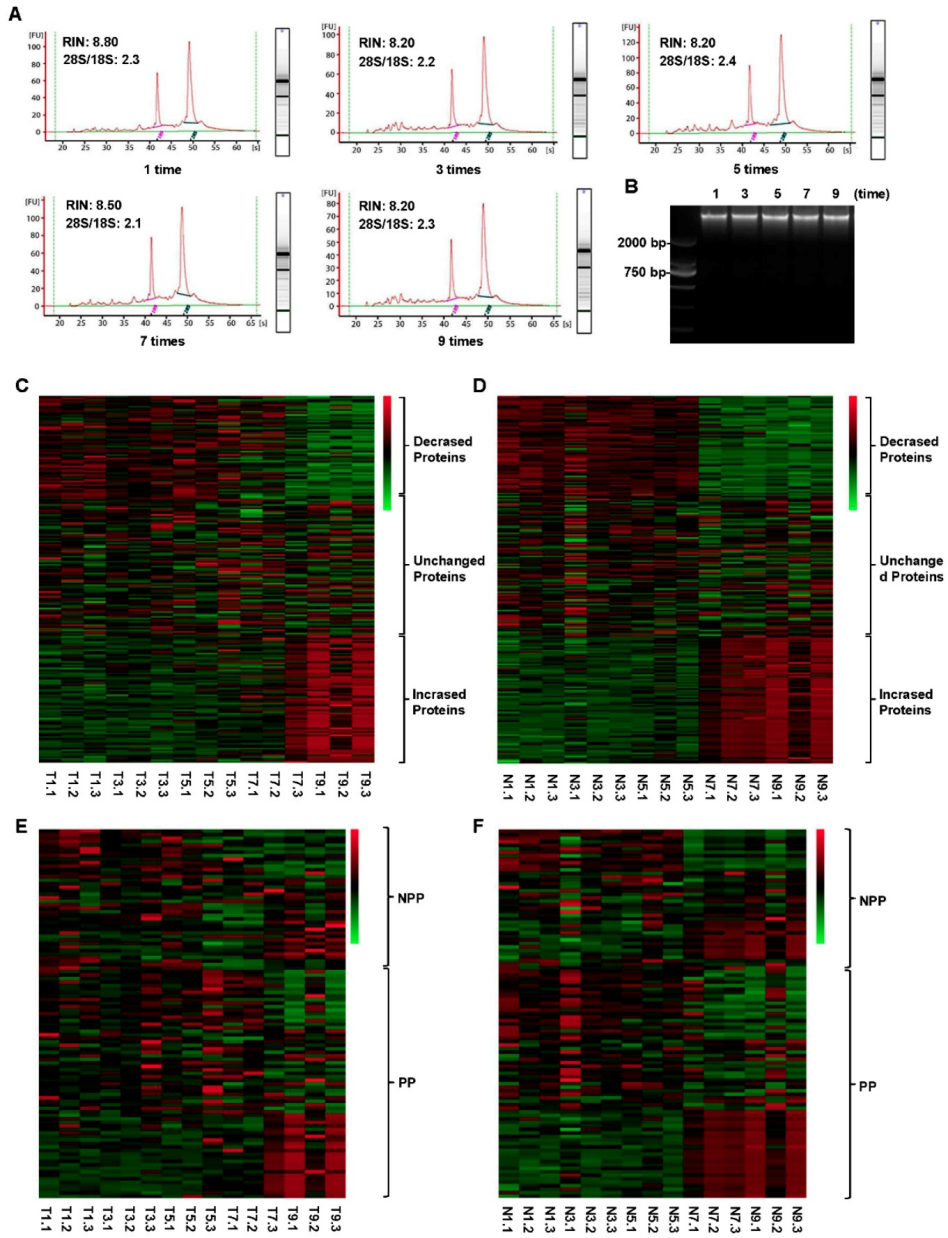
** Impact of different freeze-thaw cycles on RNA、DNA and 298 proteins integrity in the tumor and normal tissues.** The RNA (A) and DNA (B) integrity was stable in the tumor tissues even after 9 freeze-thaw cycles. The protein expression levels were obviously changed both in tumor (C) and normal tissues (D) after 7 freeze-thaw cycles. The expression pattern of part of phosphorylated proteins (PP) and non-phosphorylated proteins (NPP) was significantly changed after experienced 7 freeze-thaw cycles both in tumor (E) and normal (F) tissues. T5.2, T indicated Tumor tissue, and 5 indicated the tumor tissue experienced 5 times of freeze-thaw, and 2 indicated that the tumor tissue came from the No. 2 patient. N9.3, N indicated Normal tissue, and 9 indicated the normal tissue experienced 9 times of freeze-thaw, and 3 indicated that the normal tissue came from the No. 3 patient. The meaning of T1-9.1-3 and N1-9.1-3 can be deduced from these rules. Green color in the heatmap indicates the expression of protein is low, and red color in the heatmap indicates the protein are overexpression.

As shown in Figure 6A, all samples had high-quality RNA integrity with a RIN ≥ 7.0 during 0-9 cycles of freeze-thaw. The mean RINs were 8.7, 8.2, 8.3, 8.2 and 7.9 for 1 time, 3 times, 5 times, 7 times and 9 times of freeze-thaw in colorectal cancer tissues, respectively. Similarly, no significant alteration of DNA integrity was found during 0-9 cycles of freeze-thaw in colorectal cancer tissues (Figure 6B). For the paired normal adjacent tissues, the similar results were also observed. Therefore, up to 9 cycles, the repeated freeze-thaw had no significant effect on RNA and DNA integrity. While protein expression pattern had a significant alteration after 7 freeze-thaw cycles in both colorectal tumor tissues (Figure 6C) and normal adjacent tissues (Figure 6D). For the colorectal tumor tissues, of the 298 RPPA detected proteins, 73 proteins were decreased expressed and 84 proteins were increased expressed (Figure 6C, Table S4). For the normal adjacent tissues, 73 proteins were decreased expressed, compared to that of 84 proteins were increased expressed (Figure 6D, Table S4). Among the 65 phosphorylation proteins, 16 proteins were decreased expressed, compared to that of 21 proteins were increased expressed in tumor tissues (Figure 6E, Table S5). In normal tissues, among the 65 phosphorylated proteins, 17 phosphorylated proteins were decreased expressed, compared to that of 21 phosphorylated proteins were increased expressed (Figure 6F, Table S5). These results suggest that, to keep the fidelity of protein expression pattern, the freeze-thaw cycle of colorectal tissues should be limited to less than 7 times.

## Discussion

In the present study, we assessed the influence of cold ischemic times, temperature and freeze-thaw cycles on RNA, DNA and protein quality in colorectal tumor and normal tissues. Interestingly, we found that RNA, DNA and protein remain stable up to 48h when tissues were kept on ice after surgery. When stored at room temperature, RNA and DNA in tumor tissues were more easily to be degraded than that in normal tissues. In addition, the proteins expression levels, including of phosphorylated proteins, were significantly changed both in tumor and normal tissues when kept the tissues at room temperature for 24h. Furthermore, we found that repeated freeze-thaw cycles (up to 9 times) had no apparent effect on RNA and DNA integrity. However, proteins expression levels, including of phosphorylated proteins expression levels, were evidently changed after 7 freeze-thaw cycles. Especially, this protein expression change was obviously happened in colorectal cancer tissues.

Though the purified RNA is a labile molecule and prone to degradation, the tissue RNA seems to be more stable. A study on breast samples showed that tissue RNA remained stable for up to 24h after the surgery 11. However, the tissue RNA degradation time was varied in different studies. Micke P et al. found that the RNA in colon tissues remained stable for up to 6-16h when left at room temperature or kept on ice 12. Bao WG et al. reported that 67% of normal colon tissues and 94% of colon cancer specimens yielded high-quality RNA at 4h after the surgery 13. Here, we extended the ischemic time range from 0h to 48h, and found that RNA integrity was behaved a high quality even up to 48h when the tissues were placed on ice (Figure 2). On the other hand, the RNA degradation was detected at 8h for tumor tissues and at 24h for the paired normal adjacent tissues when kept at room temperature (Figure 3), indicating that RNA in normal adjacent tissues was more stable than colorectal cancer tissues.

With regard to the impact of freeze-thaw cycle on RNA integrity, our study showed that RNA remained intact even after 9 freeze-thaw cycles for both tumor and normal tissues (Figure 6A). Similarly, Botling J et al. also found that repetitive thawing and freezing had no detrimental effect on RNA quality as long as the total thawing time was short^14^. The potential reason should be ascribed to that RNAlater stabilization reagent prevent tissue RNA from degradation, suggesting RNA stabilization reagent should be routinely used for the tissue biobanked for RNA extraction 15.

DNA being more stable is considered better than RNA and protein for storage. Sang et al. reported that the colorectal cancer tissues DNA was stable up to 90 minutes after extraction 16. Our study found that both tumor and normal tissues DNA could keep a high quality up to 48h if the tissues were stored at on ice (Figure 4A and 4C), and even after 9 repeated freeze-thaw cycles (Figure 6B). However, the tumor tissues' DNA was degraded if the tissues were left at room temperature for 24h (Figure 4B). These results indicated that low temperature was important to keep a high quality of the tissues DNA.

Histologic evaluation of frozen tissues was often used to test the integrity of proteins 17. In terms of protein preservation, usually, sodium dodecylsulfate polyacrylamide gel electrophoresis (SDS-PAGE) coupled with Coomassie Blue staining is considered as a crude and simple approach to evaluate the integrity of protein, while, more and more previous studies had focused on the single protein assessment. The underlying reason might be that there is no effective and comprehensive way to assess the protein signature simultaneously. In this study, we used RPPA approach to investigate a large scale of protein expression. It is known that RPPA is a high-throughput technique to assess the quantitation of protein, including of phosphorylated protein, being supposed as a prominent technology in tissue quality assessment 18.

Previous studies reported that proteins, especially phosphorylated proteins, tend to be degraded, and thus rapid freezing (< 5 minutes) was critical to keep the high protein quality in tissue samples, particularly for the protein phosphorylation status 19. Compared to previous studies, we found that all of the proteins, including of phosphorylated proteins, had no remarkable changes up to 48h in both tumor and normal tissues when kept on ice (Figure 5A and 5B). However, when placed the tissue at room temperature or repeated freeze-thaw for more than 7 cycles, the proteins expression levels were dramatically altered (Figure 5E-H, Figure 6C-F). At the same time, we detected some of the 298 proteins at levels of RNA and DNA, as a result, we found that the changing trend was similar to the protein level (Data not shown).

Here, we also realized that the present study had several limitations. First, we just focused on the impact of cold ischemic time and freeze-thaw cycles on tissue RNA, DNA and protein quality. Actually, there are many other factors, such as storage time, might also affect their quality. Therefore, the other factors should be addressed in future research. Another limitation was the ischemic time. At the laparoscopic surgery era 20, the colorectal cancer operation process is standard, therefore, the tissue ischemic time is similar. However, for the bulky tumor tissues, the prolonged operation time may affect the ischemic time. This kind of clinical issues during surgical process could be the critical factor determining the quality of tissues, and should be given special attention. Our finding indicated that the colorectal tissue RNA, DNA and protein could be sustained a stable and integral status for 48h when the tissue was kept at on ice after surgery. Although we had not seen any obvious impact of 9 freeze-thaw cycles on RNA and DNA integrity, proteins expression pattern however was significantly changed after 7 freeze-thaw times. Our study provided a useful guideline to effectively keep a high quality of DNA, RNA and protein during colorectal tissue biobanking.

## Supplementary Material

Supplementary figure and tables.

## References

[1] Hewitt RE (2011). Biobanking: the foundation of personalized medicine. Curr Opin Oncol.

[2] Greely HT (2007). The uneasy ethical and legal underpinnings of large-scale genomic biobanks. Annu Rev Genomics Hum Genet.

[3] Haga SB, Beskow LM (2008). Ethical, legal, and social implications of biobanks for genetics research. Adv Genet.

[4] Schoor O, Weinschenk T, Hennenlotter J (2003). Moderate degradation does not preclude microarray analysis of small amounts of RNA. Biotechniques.

[5] Perez-Novo CA, Claeys C, Speleman F (2005). Impact of RNA quality on reference gene expression stability. Biotechniques.

[6] Hatzis C, Sun H, Yao H (2011). Effects of tissue handling on RNA integrity and microarray measurements from resected breast cancers. J Natl Cancer Inst.

[7] Liu NW, Sanford T, Srinivasan R (2013). Impact of ischemia and procurement conditions on gene expression in renal cell carcinoma. Clin Cancer Res.

[8] Bai Y, Tolles J, Cheng H (2011). Quantitative assessment shows loss of antigenic epitopes as a function of pre-analytic variables. Lab Invest.

[9] Neumeister VM, Anagnostou V, Siddiqui S (2012). Quantitative assessment of effect of preanalytic cold ischemic time on protein expression in breast cancer tissues. J Natl Cancer Inst.

[10] Jochumsen KM, Tan Q, Dahlgaard J (2007). RNA quality and gene expression analysis of ovarian tumor tissue undergoing repeated thaw-freezing. Exp Mol Pathol.

[11] Barnes RO, Parisien M, Murphy LC (2008). Influence of evolution in tumor biobanking on the interpretation of translational research. Cancer Epidemiol Biomarkers Prev.

[12] Micke P, Ohshima M, Tahmasebpoor S (2006). Biobanking of fresh frozen tissue: RNA is stable in nonfixed surgical specimens. Lab Invest.

[13] Bao WG, Zhang X, Zhang JG (2013). Biobanking of fresh-frozen human colon tissues: impact of tissue ex-vivo ischemia times and storage periods on RNA quality. Ann Surg Oncol.

[14] Botling J, Edlund K, Segersten U (2009). Impact of thawing on RNA integrity and gene expression analysis in fresh frozen tissue. Diagn Mol Pathol.

[15] Hong SH BH, Jang KY, Chung MJ (2010). Effects of delay in the snap freezing of colorectal cancer tissues on the quality of DNA and RNA. J Korean Soc Coloproctol.

[16] Johnsen IK, Hahner S, Briere JJ (2010). Evaluation of a standardized protocol for processing adrenal tumor samples: preparation for a European adrenal tumor bank. Horm Metab Res.

[17] Spurrier B, Ramalingam S, Nishizuka S (2008). Reverse-phase protein lysate microarrays for cell signaling analysis. Nat Protoc.

[18] Walker LA, Medway AM, Walker JS (2011). Tissue procurement strategies affect the protein biochemistry of human heart samples. J Muscle Res Cell Motil.

[19] Trastulli S, Cirocchi R, Listorti C (2012). Laparoscopic vs open resection for rectal cancer: a meta-analysis of randomized clinical trials. Colorectal Dis.

[20] Nelson H, Sargent DJ, Wieand HS (2004). A comparison of laparoscopically assisted and open colectomy for colon cancer. N Engl J Med.

