# Supplementary material for: Impact of Cold Ischemic Time and Freeze-Thaw Cycles on RNA, DNA and Protein Quality in Colorectal Cancer Tissues Biobanking
Source: J Cancer. 2019 Aug 27;10(20):4978–88. doi: 10.7150/jca.29372 (PMC6775519; doi:10.7150/jca.29372)
Supplement: Supplementary file 1 — Supplementary figure and tables. [file jcav10p4978s1.pdf]

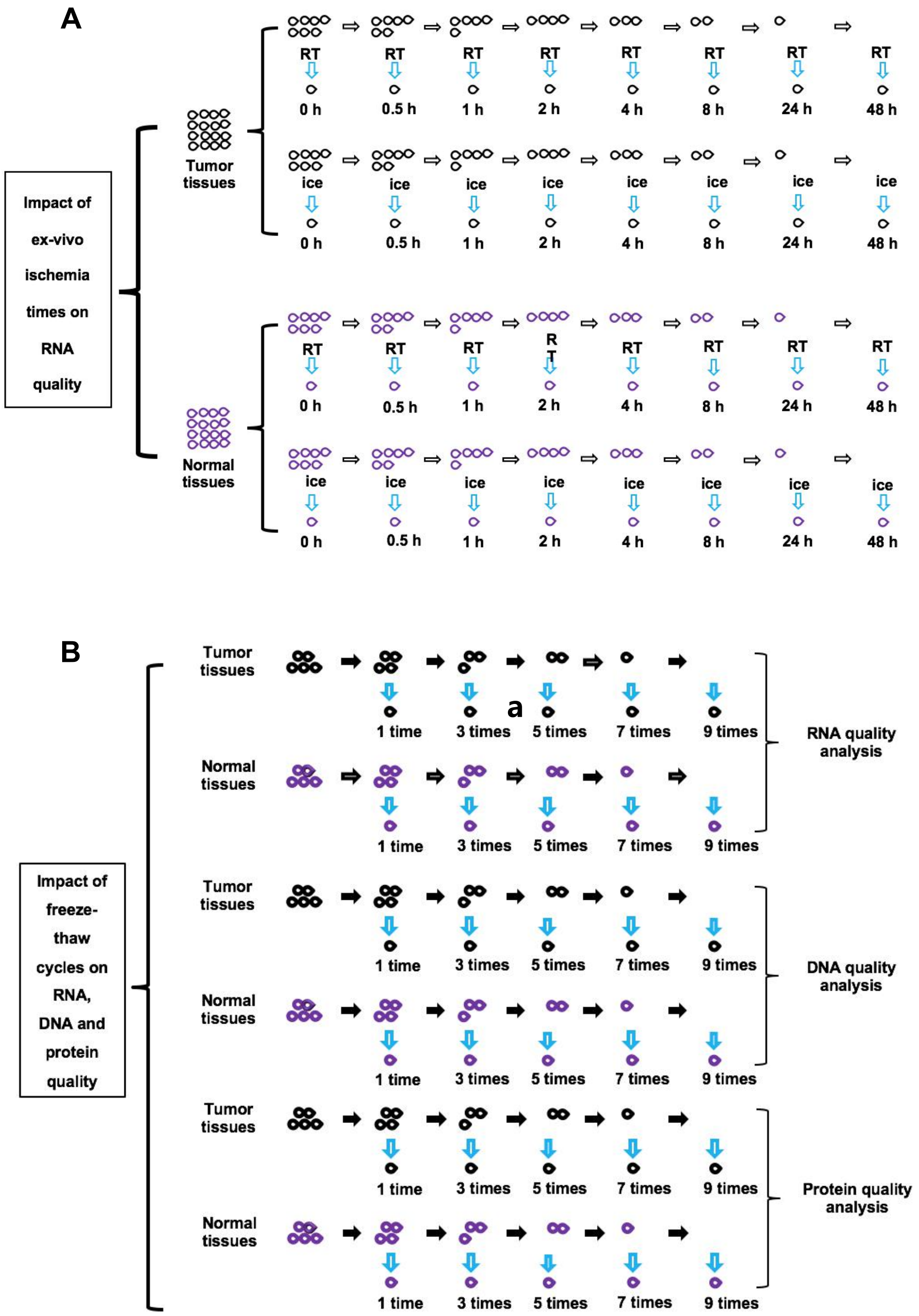

Figure S1. Study design to assess the impact of different cold ischemic times and different freeze-thaw cycles on tumor and normal tissues' quality.

Table S1. List of the proteins RPPA detected and the according antibodies used.

NO.	Official antibody Name	Antibody Company	Catalog #	Species	RPPA Dilution
1	14-3-3 beta	Santa Cruz	SC-628	Rabbit	1:75
2	14-3-3 epsilon	Santa Cruz	SC-23957	Mouse	1:50
3	14-3-3 zeta	Santa Cruz	SC-1019	Rabbit	1:5000
4	4E-BP1	CST	9452	Rabbit	1:100
5	4E-BP1 (phospho S65)	CST	9456	Rabbit	1:250
6	4E-BP1 (phospho T37/T46)	CST	9459	Rabbit	1:2000
7	53BP1	CST	4937	Rabbit	1:300
8	Acetyl CoA Carboxylase (phospho S79)	CST	3661	Rabbit	1:20000
9	Acetyl CoA Carboxylase 1	Abcam	ab45174	Rabbit	1:500
10	ACVRL1	Abcam	ab108207	Rabbit	1:30
11	ADAR1	Abcam	ab88574	Mouse	1:200
12	Akt	CST	4691	Rabbit	1:10000
13	Akt (phospho S473)	CST	9271	Rabbit	1:150
14	Akt (phospho T308)	CST	2965	Rabbit	1:500
15	AMPK alpha	CST	2532	Rabbit	1:200
16	AMPK alpha (phospho T172)	CST	2535	Rabbit	1:100
17	Androgen Receptor	Abcam	ab52615	Rabbit	1:100
18	Annexin I	BD Biosciences	610066	Mouse	1:5000
19	Annexin VII	BD Biosciences	610668	Mouse	1:30
20	A-Raf	CST	4432	Rabbit	1:150
21	ARHI	MDACC Laboratory	Bast Lab	Mouse	1:200
22	ARID1A	Sigma-Aldrich	HPA005456	Rabbit	1:1000
23	Atg3	CST	3415	Rabbit	1:750
24	Atg7	CST	8558	Rabbit	1:1000
25	ATM	CST	2873	Rabbit	1:250
26	ATM (phospho S1981)	CST	5883	Rabbit	1:25
27	ATP5A	Abcam	ab14748	Mouse	1:500
28	ATR (Phospho S428)	Abcam	ab178407	Rabbit	1:1000
29	Aurora B/AIM1	CST	3094	Rabbit	1:50
30	Axl	CST	8661	Rabbit	1:1000
31	B7-H3	CST	14058	Rabbit	1:750
32	B7-H4	CST	14572	Rabbit	1:750
33	Bad (phospho S112)	CST	9291	Rabbit	1:50
34	Bak	Abcam	ab32371	Rabbit	1:30
35	BAP1	Santa Cruz	SC-28383	Mouse	1:125
36	Bax	CST	2772	Rabbit	1:100
37	Bcl2	Dako	M0887	Mouse	1:50
38	Bcl2A1	Abnova	PAB8528	Rabbit	1:250
39	Bcl-xL	CST	2762	Rabbit	1:100
40	Beclin	Santa Cruz	SC-10086	Goat	1:250
41	beta Actin	CST	4970	Rabbit	1:75
42	beta Catenin	CST	9562	Rabbit	1:1500
43	beta Catenin (phospho T41/S45)	CST	9565	Rabbit	1:30
44	Bid	Abcam	ab32060	Rabbit	1:30
45	Bim	Abcam	ab32158	Rabbit	1:400
46	B-Raf	Abcam	ab33899	Rabbit	1:75
47	B-Raf (phospho S445)	CST	2696	Rabbit	1:1000
48	BRD4	CST	13440	Rabbit	1:200
49	c-Abl	CST	2862	Rabbit	1:50
50	Caspase-3 active	Abcam	ab32042	Rabbit	1:250
51	Caspase-7 (cleaved D198)	CST	9491	Rabbit	1:75

Table S1. List of the proteins RPPA detected and the according antibodies used.

NO.	Official antibody Name	Antibody Company	Catalog #	Species	RPPA Dilution
52	Caspase-8	CST	9746	Mouse	1:150
54	CD171 (L1)	BioLegend	826701	Mouse	1:1000
55	CD26	Abcam	ab28340	Rabbit	1:1000
56	CD29	BD Biosciences	610467	Mouse	1:30
57	CD31	Dako	M0823	Mouse	1:30
58	CD44	CST	3570	Mouse	1:50
59	CD49b	BD Biosciences	611016	Mouse	1:50
60	cdc25C	CST	4688	Rabbit	1:500
61	CDK1	Abcam	ab32384	Rabbit	1:1000
62	CDKN2A/p16INK4a	Abcam	ab81278	Rabbit	1:500
63	Chk1	CST	2360	Mouse	1:250
64	Chk1 (phospho S296)	Abcam	ab79758	Rabbit	1:125
65	Chk1 (phospho S345)	CST	2348	Rabbit	1:30
66	Chk2	CST	3440	Mouse	1:50
67	Chk2 (phospho T68)	CST	2197	Rabbit	1:125
68	c-Jun (phospho S73)	CST	9164	Rabbit	1:30
69	c-Kit	Abcam	ab32363	Rabbit	1:30
70	Claudin 7	Novus Biologicals	NB100-91714	Rabbit	1:300
71	c-Met	CST	3127	Mouse	1:250
72	c-Met (phospho Y1234/Y1235)	CST	3129	Rabbit	1:100
73	c-Myc	Santa Cruz	SC-764	Rabbit	1:125
74	COG3	ProteinTech	11130-1-AP	Rabbit	1:750
75	COL6A1	Santa Cruz	SC-20649	Rabbit	1:5000
76	Complex II Subunit	Invitrogen	459230	Mouse	1:200
77	Connexin 43	CST	3512	Rabbit	1:150
78	Coup-TFII	CST	6434	Rabbit	1:50
79	Cox IV	Abcam	ab14744	Mouse	1:50
80	Cox2	CST	4842	Rabbit	1:50
81	C-Raf (phospho S338)	CST	9427	Rabbit	1:100
82	C-Raf/Raf-1	Millipore	04-739	Rabbit	1:200
83	CXCR4	Abcam	ab2074	Rabbit	1:400
84	Cyclin B1	Epitomics	1495-1	Rabbit	1:1500
85	Cyclin D1	Santa Cruz	SC-718	Rabbit	1:200
86	Cyclin E1	Santa Cruz	SC-247	Mouse	1:30
87	Cyclophilin F	Abcam	ab110324	Mouse	1:50000
88	Cytokeratin 19	Dako	M0888	Mouse	1:50
89	Detyrosinated alpha-Tubulin	Abcam	ab48389	Rabbit	1:500
90	Dimethyl-Histone H3 (Lys4)	Millipore	07-030	Rabbit	1:1500
91	Dimethyl-K9 Histone H3	Abcam	ab32521	Rabbit	1:250
92	DUSP4/MKP2	CST	5149	Rabbit	1:250
93	Dvl3	CST	3218	Rabbit	1:30
94	E2F-1	Santa Cruz	SC-251	Mouse	1:30
95	E-Cadherin	CST	3195	Rabbit	1:300
96	eEF2	CST	2332	Rabbit	1:50
97	eEF2K	CST	3692	Rabbit	1:50
98	EGFR	CST	2232	Rabbit	1:100
99	EGFR (phospho Y1068)	CST	2234	Rabbit	1:75
100	EGFR (phospho Y1173)	Abcam	ab32578	Rabbit	1:50
101	eIF4E	CST	9742	Rabbit	1:75
102	eIF4G	CST	2498	Rabbit	1:1000
103	Elk1 (phospho S383)	CST	9181	Rabbit	1:50

Table S1. List of the proteins RPPA detected and the according antibodies used.

NO.	Official antibody Name	Antibody Company	Catalog #	Species	RPPA Dilution
104	Epithelial Membrane Antigen	Dako	M061329-2	Mouse	1:1000
105	ErbB2/HER2	Lab Vision	MS-325-P1	Mouse	1:3000
107	ErbB3/HER3	Santa Cruz	SC-285	Rabbit	1:300
108	ErbB3/HER3 (phospho Y1289)	CST	4791	Rabbit	1:50
109	ERCC1	Santa Cruz	SC-17809	Mouse	1:30
110	ERCC5	ProteinTech	11331-1-AP	Rabbit	1:250
111	ERRFI1/MIG6	Sigma-Aldrich	WH0054206M1	Mouse	1:50
112	Estrogen Receptor	Lab Vision	RM-9101	Rabbit	1:40
113	Ets-1	Bethyl	A303-501A	Rabbit	1:100
114	FAK	Abcam	ab40794	Rabbit	1:1000
115	FAK (phospho Y397)	CST	3283	Rabbit	1:30
116	Fatty Acid Synthase	CST	3180	Rabbit	1:1000
117	Fibronectin	Epitomics	1574-1	Rabbit	1:10000
118	FoxM1	CST	5436	Rabbit	1:30
119	FoxO3a	CST	2497	Rabbit	1:25
120	FoxO3a (phospho S318/S321)	CST	9465	Rabbit	1:30
121	FRA-1	Santa Cruz	SC-605	Rabbit	1:100
122	G6PD	Santa Cruz	SC-373887	Mouse	1:75
123	Gab2	CST	3239	Rabbit	1:300
124	GAPDH	Life Technologies	AM4300	Mouse	1:50000
125	GATA3	BD Biosciences	558686	Mouse	1:300
126	GCN5L2	CST	3305	Rabbit	1:30
127	Glutamate Dehydrogenase1/2	CST	12793	Rabbit	1:500
128	Glutaminase	Abcam	ab156876	Rabbit	1:250
129	Glycogen Synthase	CST	3886	Rabbit	1:1500
130	Glycogen Synthase (phospho S641)	CST	3891	Rabbit	1:250
131	GPBB	Novus Biologicals	NBP1-32799	Rabbit	1:200
132	GSK-3alpha/beta	Santa Cruz	SC-7291	Mouse	1:750
133	GSK-3alpha/beta (phospho S21/S9)	CST	9331	Rabbit	1:200
134	H2AX (phospho S140)	Pierce Biotechnology	MA1-2022	Mouse	1:400
135	Heregulin	CST	2573	Rabbit	1:30
136	HES1	CST	11988	Rabbit	1:1000
137	Hexokinase II	CST	2867	Rabbit	1:50
138	HIAP	Millipore	07-759	Rabbit	1:100
139	Hif-1 alpha	BD Biosciences	610958	Mouse	1:50
140	Histone H3	Abcam	ab1791	Rabbit	1:3000
141	HSP27	CST	2402	Mouse	1:100
142	HSP27 (phospho S82)	CST	2401	Rabbit	1:75
143	HSP70	CST	4872	Rabbit	1:150
144	IGF1R (phospho Y1135/Y1136)	CST	3024	Rabbit	1:30
145	IGFBP2	CST	3922	Rabbit	1:50
146	IGFBP5	Santa Cruz	SC-6006	Goat	1:1000
147	IGFRb	CST	3027	Rabbit	1:250
148	INPP4b	CST	4039	Rabbit	1:25
149	IRF-1	Santa Cruz	SC-497	Rabbit	1:200
150	IRS1	Millipore	06-248	Rabbit	1:400
151	JAB1	Santa Cruz	SC-13157	Mouse	1:30
152	Jagged1	Abcam	ab109536	Rabbit	1:750
153	Jak2	CST	3230	Rabbit	1:750
154	JNK/SAPK (phospho T183/Y185)	CST	4668	Rabbit	1:30
155	JNK2	CST	4672	Rabbit	1:30

Table S1. List of the proteins RPPA detected and the according antibodies used.

NO.	Official antibody Name	Antibody Company	Catalog #	Species	RPPA Dilution
156	LC3A/B	CST	4108	Rabbit	1:500
157	Lck	CST	2752	Rabbit	1:100
158	LDHA	CST	3582	Rabbit	1:250
160	Mcl 1	CST	5453	Rabbit	1:100
161	MDM2 (phospho S166)	CST	3521	Rabbit	1:50
162	MEK1	Epitomics	1235-1	Rabbit	1:1500
163	MEK1 (phospho S217/S221)	CST	9154	Rabbit	1:50
164	MEK2	CST	9125	Rabbit	1:50
165	Merlin/NF2	Novus Biologicals	22710002	Rabbit	1:250
166	MIF	Santa Cruz	SC-20121	Rabbit	1:300
167	Mitochondria	Abcam	ab3298	Mouse	1:150
168	MMP2	CST	4022	Rabbit	1:75
169	Mnk1	CST	2195	Rabbit	1:1000
170	Monocarboxylic Acid Transporter 4	Millipore	AB3314P	Rabbit	1:500
171	MSH2	CST	2850	Mouse	1:30
172	MSH6	Novus Biologicals	22030002	Rabbit	1:1000
173	mTOR	CST	2983	Rabbit	1:1000
174	mTOR (phospho S2448)	CST	2971	Rabbit	1:50
175	Myosin heavy chain 11	Novus Biologicals	21370002	Rabbit	1:5000
176	Myosin IIa (phospho S1943)	CST	5026	Rabbit	1:1000
177	Myt1	CST	4282	Rabbit	1:2000
178	NAPSIN A	Abcam	ab129189	Rabbit	1:150
179	N-Cadherin	CST	4061	Rabbit	1:30
180	NDRG1 (phospho T346)	CST	3217	Rabbit	1:100
181	NDUFB4	Abcam	ab110243	Mouse	1:30
182	NF-kappaB p65 (phospho S536)	CST	3033	Rabbit	1:30
183	Notch1	CST	3268	Rabbit	1:30
184	Notch3	Santa Cruz	SC-5593	Rabbit	1:300
185	N-Ras	Santa Cruz	SC-31	Mouse	1:50
186	p21	Santa Cruz	SC-397	Rabbit	1:150
187	p27 KIP 1	Abcam	ab32034	Rabbit	1:50
188	p27/KIP 1 (phospho T198)	Abcam	ab64949	Rabbit	1:30
189	p27/Kip1 (phospho T157)	R&D Systems	AF1555	Rabbit	1:30
190	p38 MAPK	CST	9212	Rabbit	1:1500
191	p38 MAPK (phospho T180/Y182)	CST	9211	Rabbit	1:50
192	p44/42 MAPK	CST	4695	Rabbit	1:2000
193	p53	CST	9282	Rabbit	1:2500
194	p70 S6 Kinase (phospho T389)	CST	9205	Rabbit	1:50
195	p70/S6K1	Abcam	ab32529	Rabbit	1:300
196	p90RSK (phospho T573)	CST	9346	Rabbit	1:30
197	PAI-1	BD Biosciences	612024	Mouse	1:100
198	PAR	Trevigen	4336-BPC-100	Rabbit	1:15000
199	PARK7/DJ1	Abcam	ab76008	Rabbit	1:5000
200	PARP-1	Santa Cruz	SC-7150	Rabbit	1:1500
201	Paxillin	Epitomics	1500-1	Rabbit	1:500
202	P-Cadherin	CST	2130	Rabbit	1:50
203	PCNA	Abcam	ab29	Mouse	1:200
204	Pdcd-1L1	Santa Cruz	SC-19090	Goat	1:100
205	Pdcd4	Rockland	600-401-965	Rabbit	1:750
206	PDGFR beta	CST	3169	Rabbit	1:100
207	PDK1	CST	3062	Rabbit	1:50

Table S1. List of the proteins RPPA detected and the according antibodies used.

NO.	Official antibody Name	Antibody Company	Catalog #	Species	RPPA Dilution
208	PDHK1	CST	3820	Rabbit	1:750
209	PD-L1	CST	13684	Rabbit	1:750
210	PEA-15	CST	2780	Rabbit	1:100
213	PI3K p110 beta	Santa Cruz	SC-376412	Mouse	1:60
214	PI3K p85	Millipore	06-195	Rabbit	1:15000
215	PKA RI alpha	CST	5675	Rabbit	1:200
216	PKC alpha	Millipore	05-154	Mouse	1:250
217	PKC alpha (phospho S657)	Millipore	06-822	Rabbit	1:2000
218	PKC beta II (phospho S660)	CST	9371	Rabbit	1:200
219	PKC delta (phospho S664)	Millipore	07-875	Rabbit	1:100
220	PKM2	CST	4053	Rabbit	1:300
221	PLC gamma2 (phospho Y759)	CST	3874	Rabbit	1:30
222	PLK1	CST	4513	Rabbit	1:125
223	PMS2	Novus Biologicals	22510002	Rabbit	1:1500
224	PRAS40	Invitrogen	AHO1031	Mouse	1:250
225	PRAS40 (phospho T246)	Life Technologies	441100G	Rabbit	1:500
226	PREX1	Abcam	ab102739	Rabbit	1:150
227	Progesterone Repector	Abcam	ab32085	Rabbit	1:50
228	PTEN	CST	9552	Rabbit	1:500
229	Puma	CST	4976	Rabbit	1:50
230	PYGM	Novus Biologicals	H00005837-M10	Mouse	1:500
231	Rab11	CST	3539	Rabbit	1:30
232	Rab25	CST	4314	Rabbit	1:30
233	Rad50	Millipore	05-525	Mouse	1:100
234	Rad51	CST	8875	Rabbit	1:30
235	Raptor	CST	2280	Rabbit	1:300
236	Rb	CST	9309	Mouse	1:100
237	Rb (phospho S807/S811)	CST	9308	Rabbit	1:500
238	RBM15	Novus Biologicals	21390002	Rabbit	1:5000
239	Rheb	R&D Systems	MAB3426	Mouse	1:75
240	Rictor	CST	2114	Rabbit	1:100
241	Rictor (phospho T1135)	CST	3806	Rabbit	1:200
242	Rock-1	Santa Cruz	SC-5560	Rabbit	1:1000
243	RPA32	CST	2208	Rat	1:500
244	RPA32 (Phospho S4/S8)	Bethyl	A300-245A	Rabbit	1:250
245	RSK	CST	9347	Rabbit	1:150
246	S6 (phospho S235/S236)	CST	2211	Rabbit	1:2500
247	S6 (phospho S240/S244)	CST	2215	Rabbit	1:1000
248	S6 Ribosomal Protein	CST	2317	Mouse	1:1000
249	SCD	Santa Cruz	SC-58420	Mouse	1:30
250	SDHA	CST	11998	Rabbit	1:250
251	SF2/ASF	Invitrogen	32-4500	Mouse	1:150
252	Shc (phospho Y317)	CST	2431	Rabbit	1:30
253	SHP-2 (phospho Y542)	CST	3751	Rabbit	1:75
254	SLC1A5	Sigma-Aldrich	HPA035240	Rabbit	1:15000
255	Smac/Diablo	CST	2954	Mouse	1:150
256	Smad1	Abcam	ab33902	Rabbit	1:750
257	Smad3	Abcam	ab40854	Rabbit	1:150
258	Smad4	Santa Cruz	SC-7966	Mouse	1:30
259	Snail	CST	3895	Mouse	1:50
260	SOD2	CST	13141	Rabbit	1:2500

Table S1. List of the proteins RPPA detected and the according antibodies used.

NO.	Official antibody Name	Antibody Company	Catalog #	Species	RPPA Dilution
261	Sox1	CST	4194	Rabbit	1:200
262	Sox2	CST	2748	Rabbit	1:200
263	Src (phospho Y527)	CST	2105	Rabbit	1:30
264	Src Family (phospho Y416)	CST	2101	Rabbit	1:500
265	Stat3	CST	4904	Rabbit	1:3000
266	Stat3 (phospho Y705)	CST	9131	Rabbit	1:30
267	Stat5a	Abcam	ab32043	Rabbit	1:250
268	Stathmin 1	Abcam	ab52630	Rabbit	1:75
269	Syk	Santa Cruz	SC-1240	Mouse	1:3000
270	Tau	Millipore	05-348	Mouse	1:100
271	TAZ	CST	4883	Rabbit	1:300
272	TFAM	CST	7495	Rabbit	1:300
273	TIGAR	Abcam	ab137573	Rabbit	1:100
274	Transferrin Receptor	Novus Biologicals	22500002	Rabbit	1:15000
275	Transglutaminase II	Lab Vision	MS-224-P1	Mouse	1:150
276	TSC1/Hamartin	CST	4906	Rabbit	1:200
277	TSC2/Tuberin (phospho T1462)	CST	3617	Rabbit	1:30
278	TTF1	Abcam	ab76013	Rabbit	1:30
279	Tuberin	Abcam	ab32554	Rabbit	1:2500
280	Twist	Santa Cruz	SC-81417	Mouse	1:30
281	Tyro3	CST	5585	Rabbit	1:30
282	UBAC1	Sigma-Aldrich	HPA005651	Rabbit	1:250
283	Ubiquityl Histone H2B	Millipore	05-1312	Mouse	1:500
284	UGT1A	Santa Cruz	SC-271268	Mouse	1:75
285	VDAC1/Porin	Abcam	ab14734	Mouse	1:300
286	VEGF Receptor 2	CST	2479	Rabbit	1:12000
287	VHL-EPPK1	BD Biosciences	556347	Mouse	1:1000
288	Vimentin	Dako	M0725	Mouse	1:250
289	Wee1	CST	4936	Rabbit	1:2000
290	XBP1	Santa Cruz	SC-32136	Goat	1:200
291	XIAP	CST	2042	Rabbit	1:100
292	XPA	Santa Cruz	SC-56813	Mouse	1:75
293	XPF	Abcam	ab3299	Mouse	1:100
294	XRCC1	CST	2735	Rabbit	1:30
295	YAP	Santa Cruz	SC-15407	Rabbit	1:200
296	YAP (phospho S127)	CST	4911	Rabbit	1:750
297	YB1	Novus Biologicals	17250002	Rabbit	1:750
298	YB1 (phospho S102)	CST	2900	Rabbit	1:50

Table S2. List of the decreased and increased expressed proteins in the tumor tissues and the paired normal tissues kept at room temperature for 24h after surgery

**List of the 60 decreased expressed proteins in the tumor tissues kept at room temperature for 24h after surgery**							
4E-BP1	53BP1	ACC_pS79	Akt	Annexin-I	ARID1A	Atg3	ATM
β-Catenin	Brf_pS445	Bax	BRD4	c-IAP2	Cdc25C	COG3	DUSP4
E-Cadherin	eEF2	eEF2K	eIF4E	eIF4E_pS209	eIF4G	ER	FAK_pY397
G6PD	Gab2	GSK-3a-b	HES1	HSP27_pS82	IGFBP2	MAPK_pT202_Y204	MDM2_pS166
MEK1	Mnk1	MSH6	MSI2	mTOR	NDRG1_pT346	p38-pT180-Y182	p70-S6K1
PAK1	Paxillin	Pcdc4	PKA-a	PKC-b-II_pS660	PLK1	PMS2	PTEN
Rab25	RBM15	Rictor	RIP	RSK	Src_pY527	Sky	TRIM25
Tuberin	Vimentin	YAP_pS127	ZAP-70				

**List of the 79 decreased expressed proteins in the paired normal tissues kept at room temperature for 24h after surgery**							
4E-BP1	53BP1	ACC_pS79	Akt	Annexin-I	ARID1A	Atg7	ATM
ATRX	β-Catenin	Brf_pS445	Bax	Bim	BRD4	c-IAP2	Claudin-7
COG3	Connexin-43	E-Cadherin	eEF2	eEF2K	eIF4G	EMA	FAK_pY397
G6PD	Gab2	Glutaminase	GSK-3a-b	GSK-3a-b_pS21_S9	Gys_pS641	HSP27_pS82	IGFBP2
IGFRb	JNK2	MAPK_pT202_Y204	MDM2_pS166	MEK1	MSI2	mTOR	mTOR_pS2448
Myosin-11	NDRG1_pT346	Oct-4	P27-Kip-1	p38-pT180-Y182	p70-S6K1	PAK1	Paxillin
Pcdc4	PDK1_pS241	PKA-a	PKC-a_pS657	PLK1	PMS2	PTEN	Rab25
Rad50	RBM15	RIP	RPA32	RSK	S6_pS235_S236	Shc_pY317	SHP-2_pY542
SLC1A5	Src_pY416	Src_pY527	Stat3	Stat3_pY705	Stat5a	Sky	TRIM25
Tuberin	UBAC1	VASP	VHL-EPPK1	Vimentin	YAP_pS127	ZAP-70	

<table>
<tr><td colspan="8">List of the 32 increased expressed proteins in the tumor tissues kept at room temperature for 24h after surgery</td></tr>
<tr><td>Bcl2A1</td><td>Bid</td><td>BiP-GRP78</td><td>c-Myc</td><td>Caspase-3</td><td>Caspase-7-cleaved</td><td>CD26</td><td>Collagen-VI</td></tr>
<tr><td>DJ1</td><td>DM-Histone-H3</td><td>DM-K9-Histone-H3</td><td>ERCC1</td><td>ERCC5</td><td>Ets-1</td><td>GAPDH</td><td>Heregulin</td></tr>
<tr><td>Histone-H3</td><td>LDHA</td><td>MIF</td><td>MMP2</td><td>Notch3</td><td>Oct-4</td><td>p53</td><td>PAICS</td></tr>
<tr><td>PARP1</td><td>PD-L1</td><td>PKC-delta_pS664</td><td>Rock-1</td><td>SOD1</td><td>SOD2</td><td>TFRC</td><td>Tuberin_pT1462</td></tr>
<tr><td colspan="8">List of the 47 increased expressed proteins in the normal tissues kept at room temperature for 24h after surgery</td></tr>
<tr><td>A-Raf</td><td>ATR_pS428</td><td>B7-H4</td><td>Bcl-xL</td><td>Bcl2A1</td><td>Beclin</td><td>BiP-GRP78</td><td>Caspase-3</td></tr>
<tr><td>CD26</td><td>CD49b</td><td>CDK1</td><td>Chk_pT68</td><td>Cox2</td><td>Cyclophilin-F</td><td>D-a-Tubulin</td><td>DJ1</td></tr>
<tr><td>DM-Histone-H3</td><td>DM-K9-Histone-H3</td><td>Fibronectin</td><td>FoxM1</td><td>Glutamate-D1-2</td><td>Granzyme-B</td><td>Histone-H3</td><td>HSP70</td></tr>
<tr><td>IGF1R_pY1135_Y1136</td><td>IR-b</td><td>LDHA</td><td>MIF</td><td>MIG6</td><td>MMP2</td><td>Myt1</td><td>Notch3</td></tr>
<tr><td>P-Cadherin</td><td>P16INK4a</td><td>p21</td><td>p27-pT198</td><td>p53</td><td>p70-S6K-pT573</td><td>p90RSK_pT573</td><td>PAICS</td></tr>
<tr><td>PARP1</td><td>PKC-delta_pS664</td><td>PLC-gamma2_pY759</td><td>Rab11</td><td>SOD2</td><td>Sox2</td><td>Stathmin-1</td><td></td></tr>
</table>

Table S3. List of the decreased and increased expressed phosphorylated proteins in the tumor tissues and the paired normal tissues kept at room temperature for 24h after surgery

**List of the 14 decreased expressed phosphorylated proteins in the tumor tissues kept at room temperature for 24h after surgery**							
ACC_pS79	Brf_pS445	eIF4E_pS209	FAK_pY397	FAK_pY397	HSP27_pS82	MAPK_pT202_Y204	MDM2_pS166
NDRG1_pT346	p38-pT180-Y182	p70-S6K1	PKC-b-II_pS660	Src_pY527	YAP_pS127		
**List of the 2 increased expressed phosphorylated proteins in the tumor tissues kept at room temperature for 24h after surgery**							
PKC-delta_pS664		Tuberin_pT1462					
**List of the 22 decreased expressed phosphorylated proteins in the normal tissues kept at room temperature for 24h after surgery**							
ACC_pS79	Brf_pS445	FAK_pY397	GSK-3a-b_pS21_S9	Gys_pS641	HSP27_pS82	MAPK_pT202_Y204	MDM2_pS166
mTOR_pS2448	NDRG1_pT346	P27-Kip-1	p38-pT180-Y182	p70-S6K1	PDK1_pS241	PKC-a_pS657	S6_pS235_S236
Shc_pY317	SHP-2_pY542	Src_pY416	Src_pY527	Stat3_pY705	YAP_pS127		
**List of the 7 increased expressed phosphorylated proteins in the normal tissues kept at room temperature for 24h after surgery**							
ATR_pS428	IGF1R_pY1135_Y1136	p27-pT198	p70-S6K-pT573	p90RSK_pT573	Chk_pT68	PKC-delta_pS664	

Table S4. List of the decreased and increased expressed proteins in the tumor tissues and the paired normal tissues after 7 freeze-thaw cycles

**List of the 73 decreased expressed proteins in the tumor tissues after 7 freeze-thaw cycles**							
14-3-3-beta	AMPKa	Atg7	ATM	ATM_pS1981	Aurora-B	β-Actin	β-Catenin_pT41_S45
Bak	Bax	Bcl2A1	Beclin-G	Bid	c-Kit	Caveolin-1	Chk-1_pS296
Chk2	Chk2_pT68	Claudin-7	COG3	Creb	Cyclin-D1	DJ1	E-Cadherin
E2F1	EGFR	EGFR_pY1173	eIF4E	Elk1_pS383	FoxM1	Fox03a_pS318_S321	Granzyme-B
Gys	HES1	Hexokinase-II	IGF1R_pY1135_Y1136	IGFBP2	IR-b	Lck	mTOR
p38-MAPK	p44-42-MAPK	p53	PDGFR-b	PDK-1	PDK1_pS241	PEA-15	PI3K_p85
PKC-delta_pS664	PLC-gamma2_pY759	PREX1	Rab25	Raptor	RSK	Smad1	Src
Stat3	Stat3_pY705	TRIM25	TSC1	UBAC1	VASP	YB1_pS102	14-3-3-zeta
ACC1	EMA	Myosin-11	OCT-4	PARP-1	PEA-15	TIGAR	Tuberin-pT1462
YAP_pS127							

**List of the 84 increased expressed proteins in the tumor tissues after 7 freeze-thaw cycles**							
4E-BP1_pS65	53BP1	ACC_pS79	Akt_pS473	Akt_pT308	Annexin-I	AR	ARID1A
ATRX	ATR_pS428	Axl	B-Raf	BAP1	Bcl2	BRD4	CD44
cdc25C	Chk1	Collagen-VI	Connexin-43	Cyclophilin-F	eEF2K	eIF4E_pS209	eIF4G
ENY2	ERCC5	Ets-1	FAK_pY397	Fibronectin	Gab2	GAPDH	GCLM
Glutamate-D1-2	GSK-3a-b_pS21_S9	Gys_pS641	Histone-H3	Jak2	LDHA	MCT4	MDM2_pS166
MIF	Mnk1	MSH6	MSI2	Myosin-IIa_pS1943	NAPSIN-A	NDRG1_pT346	p70-S6K1
PAK1	PAR	Paxillin	PKC-a_pS657	PKC-b-II_pS660	PKM2	PMS2	PRAS40
PRAS40_pT246	PTEN	RBM15	Rb_pS807_S811	Rheb	S6_pS235_S236	S6_pS240_S244	SLC1A5
SOD1	Src_pY416	Tau	TFRC	TUFM	ULK1_pS757	Wee1	WIPI2
Bcl-xL	Cyclin-D3	DUSP4	eEF2	FASN	FRA-1	GSK-3a-b	Hif-1-alpha
PCNA	S6	Syk	TAZ				

**List of the 73 decreased expressed proteins in the normal tissues after 7 freeze-thaw cycles**							
14-3-3-beta	ACC1	AMPKa	Atg7	ATM	ATM_pS1981	Aurora-B	b-Actin
b-Catenin_pT41_S45	Bak	Bax	Bcl2A1	Bid	c-Kit	Caveolin-1	Chk2_pT68
Claudin-7	COG3	Creb	Cyclin-D1	DJ1	E-Cadherin	E2F1	EGFR_pY1173
Elk1_pS383	EMA	FoxM1	FoxO3a_pS318_S321	Granzyme-B	Gys	HES1	Hexokinase-II
IGF1R_pY1135_Y1136	IGFBP2	IR-b	Lck	Oct-4	p38-MAPK	p44-42-MAPK	PDGFR-b
PDK1_pS241	PEA-15	PEA-15_pS116	PI3K-p85	PKC-delta_pS664	PLC-gamma2_pY759	PREX1	Rab25
Raptor	RSK	Smad1	Src	Stat3	Stat3_pY705	TIGAR	TRIM25
TSC1	UBAC1	YAP_pS127	YB1_pS102	eIF4E	p53	VASP	Myosin-11
PARP1	14-3-3-zeta	Beclin-G	Chk1_pS296	Chk2	EGFR	mTOR	PDK1
Tuberin_pT1462							

List of the 84 increased expressed proteins in the normal tissues after 7 freeze-thaw cycles							
GCLM	Glutamate-D1-2	4E-BP1_pS65	53BP1	ACC_pS79	Akt_pS473	Akt_pT308	Annexin-I
AR	ARID1A	ATRX	ATR_pS428	Axl	BAP1	Bcl-xL	BRD4
CD44	cdc25C	Chk1	Collagen-VI	Connexin-43	Cyclophilin-F	DUSP4	eEF2
eEF2K	eIF4E_pS209	eIF4G	ENY2	ERCC5	Ets-1	FAK_pY397	FASN
Fibronectin	FRA-1	Gab2	GAPDH	GSK-3a-b_pS21_S9	Gys_pS641	Hif-1-alpha	Jak2
LDHA	MCT4	MDM2_pS166	MIF	Mnk1	MSH6	MSI2	Myosin-IIa_pS1943
NAPSIN-A	NDRG1_pT346	p70-S6K1	PAK1	PAR	Paxillin	PCNA	PKC-a_pS657
PKC-b-II_pS660	PKM2	PMS2	PRAS40	PRAS40_pT246	PTEN	RBM15	Rb_pS807_S811
Rheb	S6	S6_pS235_S236	S6_pS240_S244	SLC1A5	SOD1	Src_pY416	Syk
Tau	TAZ	TFRC	ULK1_pS757	Wee1	WIPI2	B-Raf	Bcl2
Cyclin-D3	GSK-3a-b	Histone-H3	TUFM				

Table S5. List of the decreased and increased expressed phosphorylated proteins in the normal tissues and the paired normal tissues after 7 freeze-thaw cycles

**List of the 16 decreased expressed phosphorylated proteins in the normal tissues after 7 freeze-thaw cycles**							
ATM_pS1981	β-Catenin_pT41_S45	Chk-1_pS296	Chk2_pT68	EGFR_pY1173	Fox03a_pS318_S321	IGF1R_pY1135_Y1136	Elk1_pS383
PDK1_pS241	PI3K_p85	PKC-delta_pS664	PLC-gamma2_pY759	Stat3_pY705	YB1_pS102	Tuberin-pT1462	YAP_pS127
**List of the 21 increased expressed phosphorylated proteins in the normal tissues after 7 freeze-thaw cycles**							
4E-BP1_pS65	ACC_pS79	Akt_pS473	Akt_pT308	ATR_pS428	eIF4E_pS209	FAK_pY397	GSK-3a-b_pS21_S9
Gys_pS641	MDM2_pS166	Myosin-IIa_pS1943	NDRG1_pT346	p70-S6K1	PKC-a_pS657	PKC-b-II_pS660	PRAS40_pT246
Rb_pS807_S811	S6_pS235_S236	S6_pS240_S244	Src_pY416	ULK1_pS757			
**List of the 17 decreased expressed phosphorylated proteins in the normal tissues after 7 freeze-thaw cycles**							
ATM_pS1981	b-Catenin_pT41_S45	Chk2_pT68	EGFR_pY1173	Elk1_pS383	FoxO3a_pS318_S321	IGF1R_pY1135_Y1136	PDK1_pS241
PEA-15_pS116	PI3K-p85	PKC-delta_pS664	PLC-gamma2_pY759	Stat3_pY705	YAP_pS127	YB1_pS102	Chk1_pS296
Tuberin_pT1462							
**List of the 21 increased expressed phosphorylated proteins in the normal tissues after 7 freeze-thaw cycles**							
4E-BP1_pS65	ACC_pS79	Akt_pS473	Akt_pT308	ATR_pS428	eIF4E_pS209	FAK_pY397	GSK-3a-b_pS21_S9
Gys_pS641	MDM2_pS166	Myosin-IIa_pS1943	NDRG1_pT346	p70-S6K1	PKC-a_pS657	PKC-b-II_pS660	PRAS40_pT246
Rb_pS807_S811	S6_pS235_S236	S6_pS240_S244	Src_pY416	ULK1_pS757			
